# Rare and frequent postoperative complications after combined pneumonectomy for nonsmall-cell lung cancer

**DOI:** 10.1186/1749-8090-8-S1-P134

**Published:** 2013-09-11

**Authors:** N Lyas, V Khurnin, S Pushkin, A Benyan

**Affiliations:** 1 Samara Regional Oncological Hospital, Samara, Russian Federation; 2 Samara Regional Clinical Hospital, Samara, Russian Federation

## Background

The rate and structure of postoperative complications after combined pneumonectomy for non small-cell lung cancer depends not only from character of disease, but also because of features of performing operations.

## Methods

The study includes 686 patients who underwent pneumonectomy for primary non-small cell lung cancer (NSCLC) from 2003 through 2012. There were male – 609 (88, 8%), female – 77 (11,2%). Squamous cell and adenocarcinoma were found in 492 (72%) and 145 (21%) patients, respectively. Right side pneumonectomy was performed at 337 patients, left side – at 349 patients. The combined pneumonectomy was performed at 144 patients (21%): with pericardial resection – 75, wedge carinal resection – 40, circular carinal resection – 12, resection of upper cava vein – 13, of esophagus – 7, of vagus - 7, of costa – 5, of left atrium – 3, of diaphragm – 2, pleurectomy – 2, resection of aortic wall – 1.

## Results

In structure of complications the most frequent was bronchopleural fistula – 29 patients (4, 2%), mortality was – 34, 5%. Right side fistula observed in 3 times more often than left sided. After carinal resection frequency of bronchopleural fistula was 9, 6%, mortality – 80%. Another complications were: cardiorespiratory insufficiency – 9 patients (1, 3%), intrapleural bleeding – 8 (1, 2%), hemothorax - 3(0,4%), pulmonary embolism - 6(0,9%), cerebral stroke - 3(0,4%). Rare complications, those we have also observed, were: cardiac dislocation occurred after intrapericardial right pneumonectomy with extended pericardioectomy (1 patient), chylothorax (1 patient), mesenteric thrombosis (1 patient), and spontaneous rupture of the esophagus (1 patient). The general rate of complications was 9,5%, mortality – 4,2%.

## Conclusions

So, bronchopleural fistula is the most frequent complication after combined pneumonectomy for nonsmall-cell lung cancer. Variety of other complications depends from severity of disease and volume of primary operation.

